# Induction of KSHV latency-associated nuclear antigen (LANA) by hypoxia and hypoxia-inducible factors (HIF)

**DOI:** 10.1186/1750-9378-7-S1-P37

**Published:** 2012-04-19

**Authors:** Ravindra P Veeranna, Muzammel Haque, David A Davis, Min Yang, Robert Yarchoan

**Affiliations:** 1 HIV and AIDS Malignancy Branch, Center for Cancer Research, National Cancer Institute, National Institutes of Health, Bethesda, MD, USA

## 

Hypoxia activates KSHV lytic replication in primary effusion lymphoma (PEL) cells. In the current study, we show that LANA mRNA levels were up regulated in BC-3 PEL cells in hypoxia. Further, the total levels of LANA protein were elevated in BC-3 PEL cells after 24 h hypoxia and increased through 72 h. Also, infection of BC3 cells with a retroviral vector encoding siRNA to HIF-2α (or HIF-1α) decreased the levels of LANA protein. By contrast, protein levels of LANA-2, which is also a latent protein, remained unchanged in hypoxia. Computer analysis of a 1.2-kb sequence upstream of the LANA translational start site revealed six potential hypoxia-responsive elements (HRE). Reporter assays in Hep3B cells utilizing this region resulted in moderate activation by hypoxia and CoCl2 (a hypoxia mimic) and greater activation by co-transfection with degradation-resistant HIF-1α or HIF-2α. Greater induction was seen with HIF-2α than HIF-1α. Sequential deletion studies revealed that much of this activity was mediated by one of these HREs (HRE 4R) oriented in the 3’ to 5’ direction located between the constitutive (LTc) and RTA-inducible (LTi) mRNA start sites. Site directed mutation of this HRE substantially reduced the response to both HIF-1α and HIF-2α in reporter assays. Electrophoretic mobility shift assays (EMSA) and chromatin immunoprecipitation (ChIP) assays demonstrated binding of both HIF-1α and HIF-2α to this region. Consistent with the reporter assays, ChIP revealed greater binding of HIF-2α than HIF-1α. These observations suggest that hypoxia induces the transcriptional activation of LANA by the interaction of HIF through at least one HRE in the LANA promoter region and that this activity is preferentially responsive to HIF-2α. Computer analysis of LTi promoter revealed the presence of RTA-responsive elements adjacent to HRE 4R and 5R. Cotransfection assays in Hep3B cells revealed that RTA cooperates with HIF to induce LTi promoter activity. Hypoxia or CoCl2 treatment of Hep3B cells transfected with RTA confirmed this cooperative effect on LTi promoter activity. Immunoprecipitation assays using the nuclear extract of PEL cells exposed to hypoxia revealed that RTA associates with HIF-1α and HIF-2α to activate the inducible LANA promoter. Taken together with previous studies, these results provide evidence that hypoxia and HIFs activate both latent and lytic KSHV replication and play a central role in the life cycle of this virus.

